# 

**Published:** 1900-08

**Authors:** 


**August, 1900-July, 1901.**

---

| | |
|---|---|
| Bainbridge, William Seaman | New York. |
| Ballou, E. H. | Gardenville, N. Y. |
| Bayliss, A. W. | Buffalo. |
| Beahan, A. L. | Canandaigua. |
| Benedict, A. L. | Buffalo. |
| Bennett, M L | Watkins, N. Y. |
| Bissell, William G. | Buffalo. |
| Busch, F. C. | Buffalo. |
| Carstens, J. Henry | Detroit. |
| Chase, Walter B. | New York. |
| Cheesman, William S. | Auburn. |
| Church, W. F. | Chicago. |
| Clark, L. Pierce | Sonyea, N. Y. |
| Clinton, Marshall | Buffalo. |
| Cott, George F. | Buffalo. |
| Congdon, Charles E. | Buffalo. |
| Crockett, Montgomery A. | Buffalo. |
| Daggett, Byron H. | Buffalo. |
| Dickinson, William L. | Saginaw. |
| Dowd, J. Henry | Buffalo. |
| Dwyer, Thomas F. | Buffalo. |
| Filsinger, F. | Buffalo. |
| Frye, Maud J | Buffalo. |
| Fronczak, Francis E. | Buffalo. |
| Gram, F. C. | Buffalo. |
| Gaylord, Harvey R. | Buffalo. |
| Goler, George W. | Rochester. |
| Hanley, L. G. | Buffalo. |
| Hinkel, Frank Whitehill | Buffalo. |
| Hopkins, Henry R. | Buffalo. |
| Howe, Lucien | Buffalo. |
| Howe, William A. | Phelps, N. Y. |
| Hubbell, A. A. | Buffalo. |
| Hurd, Arthur W. | Buffalo. |
| Jack, George N. | Depew, N. Y. |
| Jacobson, Nathan | Syracuse. |
| Johnson, J. Roberts | Syracuse. |
| Kenerson, Vertner | Buffalo. |
| Kerr, A. T. | Ithaca. |

### CONTRIBUTORS TO VOLUME XL.

| | |
|---|---|
| King, Clarence | Machias, N. Y. |
| Krauss, William C. | Buffalo. |
| Lake, A. D. | Gowanda, N. Y. |
| LeBreton, Prescott | Buffalo. |
| Long, B. G. | Buffalo. |
| Lyon, Irving P. | Buffalo. |
| Maloney, Frank W. | Rochester. |
| Mann, Matthew, D | Buffalo. |
| Matzinger, H. G. | Buffalo. |
| McGuire, F. W. | Buffalo. |
| Meyer, P. H. | Buffalo. |
| Millener, Frederick H. | Buffalo. |
| Mynter, Herman | Buffalo. |
| O'Gorman, Francis M. | Buffalo. |
| Park Roswell. | Buffalo. |
| Potter, William Warren | Buffalo. |
| Putnam, James W. | Buffalo. |
| Rasbach, F. B. | Buffalo. |
| Ravenal, Mazyck P. | Philadelphia. |
| Renner, W. Scott | Buffalo. |
| Richy, H. A. | New York. |
| Ring, William G. | Buffalo. |
| Ruggles, E. Wood | Rochester. |
| Satterlee, Richard H. | Buffalo |
| Smith, A. Everitt | Olean, N. Y. |
| Smith, Eugene A | Buffalo. |
| Smith, Chauncey Pelton | Buffalo. |
| Stanton, William | Webster, N. Y. |
| Stephenson, F. H. | Syracuse. |
| Stevens, George T. | New York. |
| Ullman, Julius | Buffalo. |
| Vander Beek, C. A. | Rochester. |
| Veeder, M. A. | Lyons, N. Y. |
| Wende, Ernest | Buffalo. |
| Wende, Grover W. | Buffalo. |
| Whitford, William | Chicago. |
| Wilson, Nelson W. | Buffalo. |
| Wright, Alfred B. | Buffalo. |
| Young, A. A. | Newark, N. Y. |

Buffalo Academy of Medicine.

Clinical Society, Buffalo General Hospital.

Medical Association of Central New York.

Medical Society of the County of Erie.

### BOOKS RECEIVED.

A Manual of Operative Surgery. By Lewis A. Stimson, B. A., M. D., Professor of Surgery in Cornell University Medical College. New (4th) and thoroughly revised edition. In one royal 12mo volume of 581 pages with 293 illustrations. Just ready Cloth, $3.00 net. Philadelphia and New York: Lea Brothers & Co. 1900.

Diseases of the Eye. By Edward Nettleship, F. R. C. S., Ophthalmic Surgeon at St. Thomas' Hospital, London; Surgeon to the Royal London Ophthalmic Hospital, etc. New (6th) American from the sixth English edition. Thoroughly revised by William Campbell Posey, M. D. With a supplement on the detection of color blindness by William Thomson, M. D, Professor of Ophthalmology in the Jefferson Medical College, Philadelphia. Just ready. In one 12mo volume of 562 pages, with 192 illustrations. Selections from Snellen's test-types and formulæ and 5 colored plates. Cloth, $2.25 net. Philadelphia and New York: Lea Brothers & Co. 1900.

A Manual of Surgical Treatment. By W. Watson Cheyne, M. B., F. R. C. S., F. R. S., Professor of Surgery in King's College, London; Surgeon to King's College Hospital, etc. And F. F. Burghard, M. D. and M. S. (Lond.), F. R. C. S., Teacher of Practical Surgery in King's College, London; Surgeon to King's College Hospital, etc. In seven imperial 8vo volumes, with illustrations. Volume III., 305 pages, with 100 illustrations. Cloth, $3.50, net. Philadelphia and New York: Lea Brothers & Co. 1900.

Fractures. By Carl Beck, M. D., Visiting Surgeon to St. Mark's Hospital and to the New York German Poliklinik. With an appendix on the Röntgen Rays. Octavo, pp. 335; 178 illustrations. Price, $3.00, net. Philadelphia: W. B. Saunders & Co. 1900.

Essentials of Medical and Clinical Chemistry. With Laboratory Exercises. By Samuel E. Woody, A. M., M. D. Fourth edition, revised and enlarged. Illustrated. Price, $1.50. Philadelphia: P. Blakiston's Son & Co. 1900.

A Treatise on Appendicitis. By John B. Deaver, M. D., Surgeon-in-Chief to the German Hospital, Philadelphia. Second edition, Thoroughly revised and considerably enlarged. Illustrated, with 32 full-page plates. Octavo, $3.50, net. Philadelphia: P. Blakiston's Son & Co. 1900.

Food for the Sick and How to Prepare It. With a Chapter on Food for the Baby. By Edwin Charles French, M. D. Price, $1.00. Louisville: John P. Morton & Co. 1900.

A Manual of Clinical Diagnosis by Microscopical and Chemical Methods. For Students, Hospital Physicians and Practitioners. By Charles E. Simon, M. D., Late Assistant Resident Physician Johns Hopkins Hospital, Baltimore. In one very handsome 8vo volume of 563 pages, with 136 engravings and 18 full-page colored plates. Cloth, $3.50, net. Philadelphia and New York: Lea Brothers & Co. 1900.

A Dictionary of Medicine and the Allied Sciences. Comprising the Pronunciation, Derivation and Full Explanation of Medical, Pharmaceutical, Dental and Veterinary Terms; together with much collateral descriptive matter, numerous tables, etc. By Alexander Duane, M. D., Assistant Surgeon to the New York Ophthalmic and Aural Institute; Reviser of Medical Terms for Webster's International Dictionary. New (3d) edition. In one large square 8vo volume of 656 pages, with 8 full-page colored plates. Cloth, $3.00, net; full flexible leather, $4.00, net. Philadelphia and New York: Lea Brothers & Co. 1900.

A Text-Book of Practical Medicine. By William Gilman Thompson, M. D., Professor of Medicine in Cornell University Medical College, New York City, Physician to the Presbyterian and Bellevue Hospitals, New York. In one magnificent 8vo volume of 1010 pages, with 79 engravings. Cloth, $5.00, net; leather, $6.00, net; half Morocco, $6.50, net. Lea Brothers & Co., Publishers. 1900.

Transactions of the Associated Physicians of Long Island. June, 1898, to January, 1900. Volume I. Edited by Robert J. Morrison, M. D.

Transactions of the Medical Association of Central New York. Thirty-second annual meeting, held at Syracuse, N. Y., October, 1899. Wm. C. Krauss, M. D., Editor. Volume VI. Buffalo: Buffalo Medical Journal Print. 1900.

Transactions of the American Association of Obstetricians and Gynecologists. Volume XII. For the year 1899. Edited by William Warren Potter, M. D., Secretary. Philadelphia: W. J. Dornan, Printer. 1900.

